# Indoor Group Identification and Localization Using Privacy-Preserving Edge Computing Distributed Camera Network

**DOI:** 10.1109/jispin.2024.3354248

**Published:** 2024-01-16

**Authors:** Chaitra Hegde, Yashar Kiarashi, Amy D. Rodriguez, Allan I. Levey, Matthew Doiron, Hyeokhyen Kwon, Gari D. Clifford

**Affiliations:** Georgia Institute of Technology, Atlanta, GA 30332 USA; Emory University, Atlanta, GA 30322 USA; Emory University, Atlanta, GA 30322 USA; Emory University, Atlanta, GA 30322 USA; Emory University, Atlanta, GA 30322 USA; Emory University, Atlanta, GA 30322 USA; Georgia Institute of Technology, Atlanta, GA 30332 USA; Emory University, Atlanta, GA 30322 USA

**Keywords:** Cameras, group position detection, group position estimation, pose estimation

## Abstract

Social interaction behaviors change as a result of both physical and psychiatric problems, and it is important to identify subtle changes in group activity engagements for monitoring the mental health of patients in clinics. This work proposes a system to identify when and where group formations occur in an approximately 1700 m^2^ therapeutic built environment using a distributed edge-computing camera network. The proposed method can localize group formations when provided with noisy positions and orientations of individuals, estimated from sparsely distributed multiview cameras, which run a lightweight multiperson 2-D pose detection model. Our group identification method demonstrated an F1 score of up to 90% with a mean absolute error of 1.25 m for group localization on our benchmark dataset. The dataset consisted of seven subjects walking, sitting, and conversing for 35 min in groups of various sizes ranging from 2 to 7 subjects. The proposed system is low-cost and scalable to any ordinary building to transform the indoor space into a smart environment using edge computing systems. We expect the proposed system to enhance existing therapeutic units for passively monitoring the social behaviors of patients when implementing real-time interventions.

## I. Introduction

Humans are social beings and social interactions are an important part of human life. Studies have shown a link between healthy social interactions and low rates of depression [1], [2]. The recent COVID-19 pandemic also highlighted the positive impact of social interactions on work effectiveness [3]. A decrease in social engagements can be an indication of mental health problems, such as depression [4], schizophrenia [5], or cognitive impairment [6]. Hence, techniques that can quantify social interaction behaviors of individuals, such as frequency, duration, or degree of engagement in indoor environments, can provide critical insights into mental and cognitive illnesses, where early detection and intervention can change the disease prognosis [7].

Group detection has been an active research topic with increase in interest in social signal processing [8], [9], [10]. However, implementing a scalable, passive monitoring system that can continuously track group formation and interaction in a therapeutic environment across a large area is technically intractable. Previously, for localizing detailed movements during indoor activities, multiple studies required significant modifications to existing infrastructures [11]. They proposed to densely install wireless proximity sensors, such as radio frequency identification (RFID), Bluetooth beacons, WiFi access points, or ultrawideband beacons across the indoor space every 20 cm–1 m [12], [13], [14]. Those approaches also required subjects to carry an active tag, like RFID, to track their movements.

With the advancements in computer vision techniques, many works have proposed detecting social groups from cameras to understand the social relationships between the subjects [15], [16], [17]. Choi et al. [18] proposed a bottom-up approach to localize groups of people frame-by-frame from single camera images, first detecting people using the pose estimation technique, which is then clustered based on the geometrical configuration of multiperson poses. Others also proposed detecting groups in videos based on multiperson tracking outputs. Some proposed applying graph-based clustering to detect groups from a set of tracklets [19], [20], or learning-based approach to detect pairwise interaction between pedestrians using position and head pose features [21]. Although effective, those proposed methods required subjects to be within the field of view to detect and track groups across frames.

Other studies have used cameras placed at elevated positions to detect group formations by analyzing the positions and head orientations of individuals. Cristani et al. [8] and Setti et al. [22] used a voting strategy based on Hough transform to interpret positions and facing orientations for group detection. Hung and Kröse [16] used the concept of dominant sets in top–down camera images to identify interacting groups. However, these approaches were not concerned with the privacy of occupants in the indoor space and obtained the positions and head orientations of individuals from raw image frames. Moreover, they relied on a small number of cameras to monitor a small area, often having significantly large overlapping viewpoints. Furthermore, while noise in estimated positions and orientations of individuals has been accounted for in some of these group detection works, the real-world scenarios having missed detections (false negatives) or false detections (false positives) of individuals have not been sufficiently studied. Compared to previous work, the proposed work demonstrates that group localization and identification are feasible when deploying multiple cameras sparsely in wide indoor spaces in the real world while maintaining user privacies.

Multiperson localization and camera tracking techniques are still an unsolved problem undergoing active research [23]. Especially for therapeutic units, where complex movements and trajectories are observed by both staff and patients, perfect detection and localization of multiple people in potentially crowded and dynamic environments is likely not true. This calls for an alternative approach, which can detect and localize social groups when presented with noisy positions and orientation of individuals in the space. In this work, we propose a pipeline to identify the occurrence of group formations and estimate their locations using a distributed edge-computing camera network in a built environment with an emphasis on the privacy of individuals. In contrast to prior studies, which assume cleanly estimated positions or orientations of the people in the field of view, we demonstrate the feasibility of achieving robust group identification and localization in a real-world setting, where privacy-preserving, low-cost, distributed edge computing systems capture noisy positions and orientations of individuals spanning a substantially large area.

Our study is conducted within an expansive 1700 m^2^ therapeutic facility involving both patients and staff, which serves as our primary research site. We refer to this site as the *study site* in the rest of this document. Our primary objective is to gauge social interactions by identifying group formations, a process we refer to as “group identification,” while simultaneously pinpointing the precise locations of these groups, which we term “group localization” [24]. To accomplish this, we propose a scalable approach to transform ordinary therapeutic units to passively track group interaction behaviors using edge computing systems. Our approach builds upon the foundation laid by Kwon et al. [25], whose pioneering work involves the continuous localization and tracking of multiple individuals within a sprawling indoor space characterized by a complex layout. This is achieved through the use of a privacy-preserving, sparsely distributed camera network.

To test the proposed method, we collected a benchmark dataset for group formation with seven participants across different spaces in our study site. We obtained an F1 score of up to 90% for group identification and a mean absolute error (MAE) of 1.25 m for group localization using the proposed method. Our detailed analysis demonstrated differences in group detection performance depending on the camera layouts capturing the space locally. While the framework presented is generalizable to various contexts, this study was conducted in the study site specifically designed for offering therapeutic activities and opportunities for social interaction to individuals with mild cognitive impairment (MCI), a condition characterized by cognitive decline mostly seen in older adults [26].

## II. Method and Materials

The proposed group identification and localization system is built from the distributed camera and edge computing system deployed in the therapeutic study space used by individuals with cognitive impairment [25]. In this section, we will first discuss a comprehensive overview of the study space and the deployed edge computing system. Afterward, we introduce our group identification and localization methodology.

### A. Study Site

**Fig. 1.**
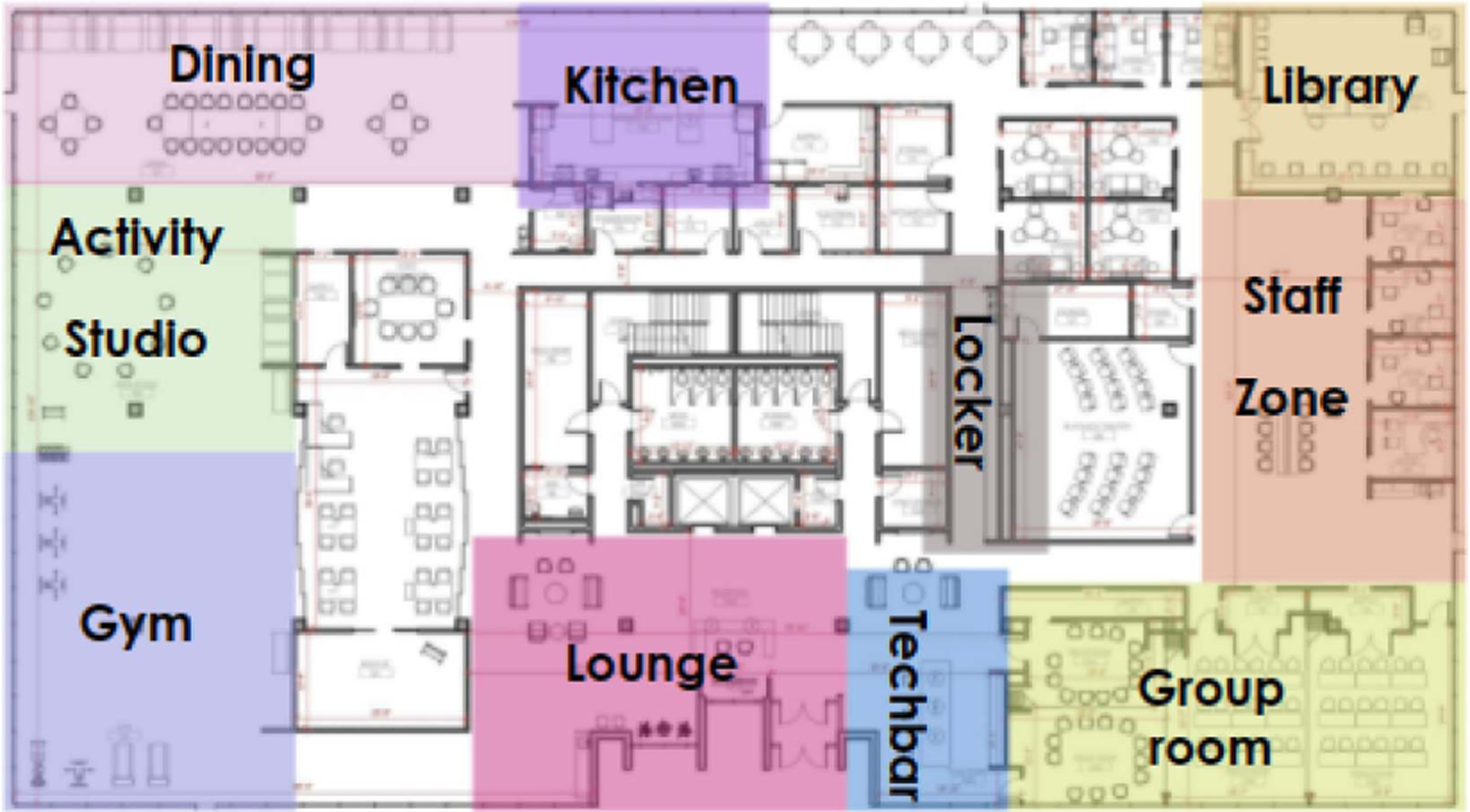
Layout of the indoor space spanning 1700 m^2^ in our study. Our study site has various regions to provide physical and cognitive training for individuals with mild cognitive impairment relating to activities in daily living. These areas include a gym, dining area, kitchen, lounge, activity area, tech bar, and staff zone. The proposed work could successfully identify and localize social groups across these regions.

The research conducted in this work took place within a specially designed indoor environment spanning 1700 m^2^. This environment comprises various functional spaces, including a library, kitchen, dining area, gym, and more, as illustrated in Fig. 1. The purpose of this unique site is to enhance the lives of individuals with MCI. It stands as a one-of-a-kind project that aims to achieve several goals: promoting social interaction, facilitating the exploration of the surroundings, offering spatial flexibility, ensuring safety, enabling independent learning of everyday tasks like cooking, incorporating a connection with nature, encouraging physical exercise, and providing cognitive stimulation for those diagnosed with MCI. The spaces within this study site are designed to be adjustable and are equipped with tunable lighting and controllable sound systems, which support achieving the aforementioned goals.

### B. Position and Orientation Estimation Using Edge Computing Systems

**Fig. 2.**
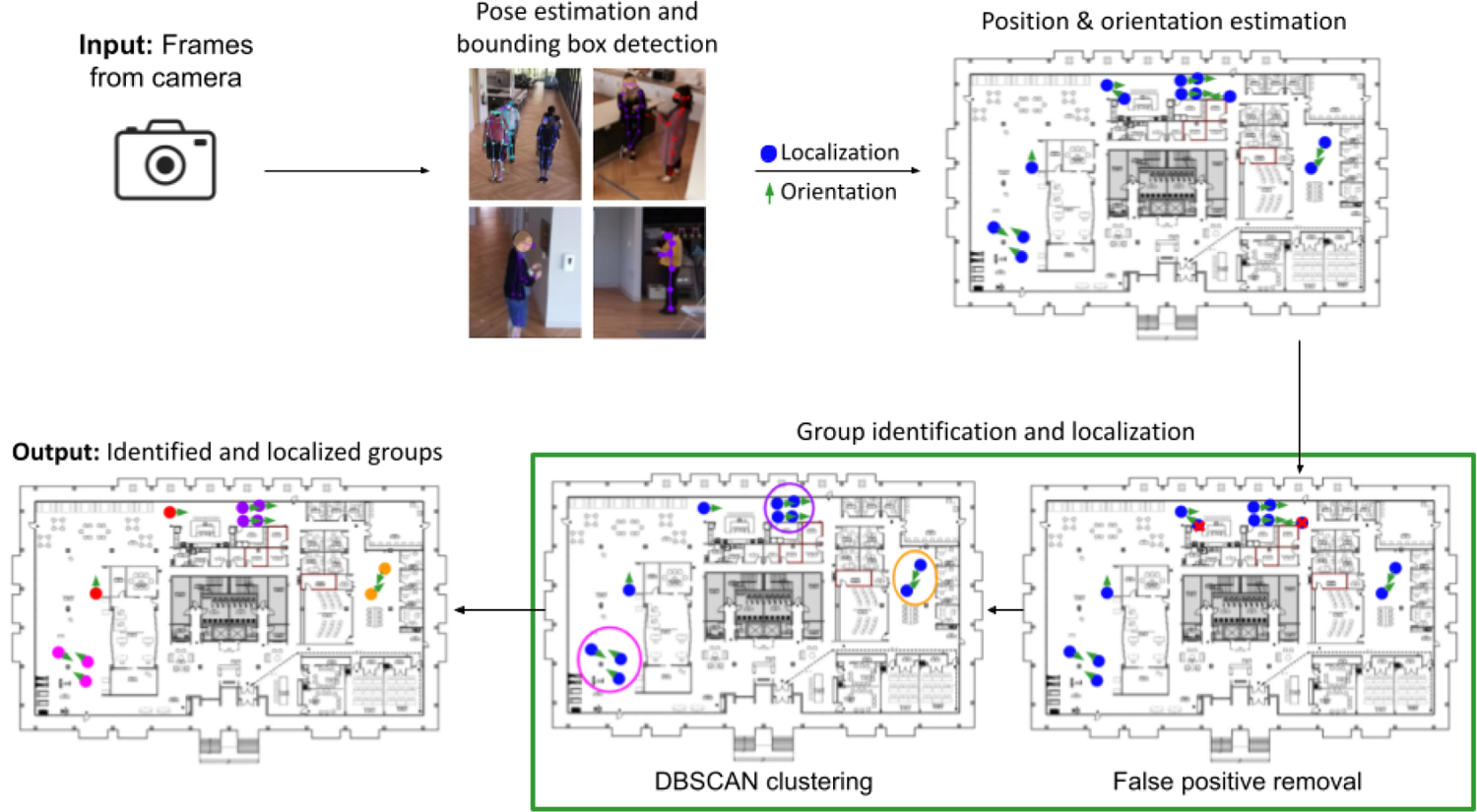
Overall pipeline for group identification and localization. The frames obtained by the cameras are processed in real time on the edge computing camera device to obtain the poses and bounding boxes of individuals in the study space. These are used to estimate the positions and facing orientations of individuals in the space. The false positives in the estimated positions and orientations are removed following which groups are identified and localized using DBSCAN. This figure is borrowed from Kwon et al. [25].

The proposed work aims to identify and localize social groups in a real-world scenario where multiperson detection and localization potentially include false positives (human ghosts) and false negatives (missed detections). The pipeline of our system has three major parts, as shown in Fig. 2. First, the edge devices estimate the 2-D poses and bounding boxes of individuals captured by the cameras in real time at a frame rate of 1 Hz at the point of image capture. These are processed to localize, track, and estimate body orientations of individuals while performing group activities [25]. The estimated locations and body orientations from all cameras are aggregated to handle the overlapping fields of view of cameras, ensuring that each individual is assigned only one estimate of position and orientation. All computations to estimate locations, orientations, and trajectories, and to aggregate multiple camera views take place on a computational server where the 2-D poses and bounding boxes are stored for all cameras. Then, the detected positions and body orientations are used to identify and localize social groups among individuals. Here, we will discuss our pipeline in more detail.

#### 1) Camera Installation:

**Fig. 3.**
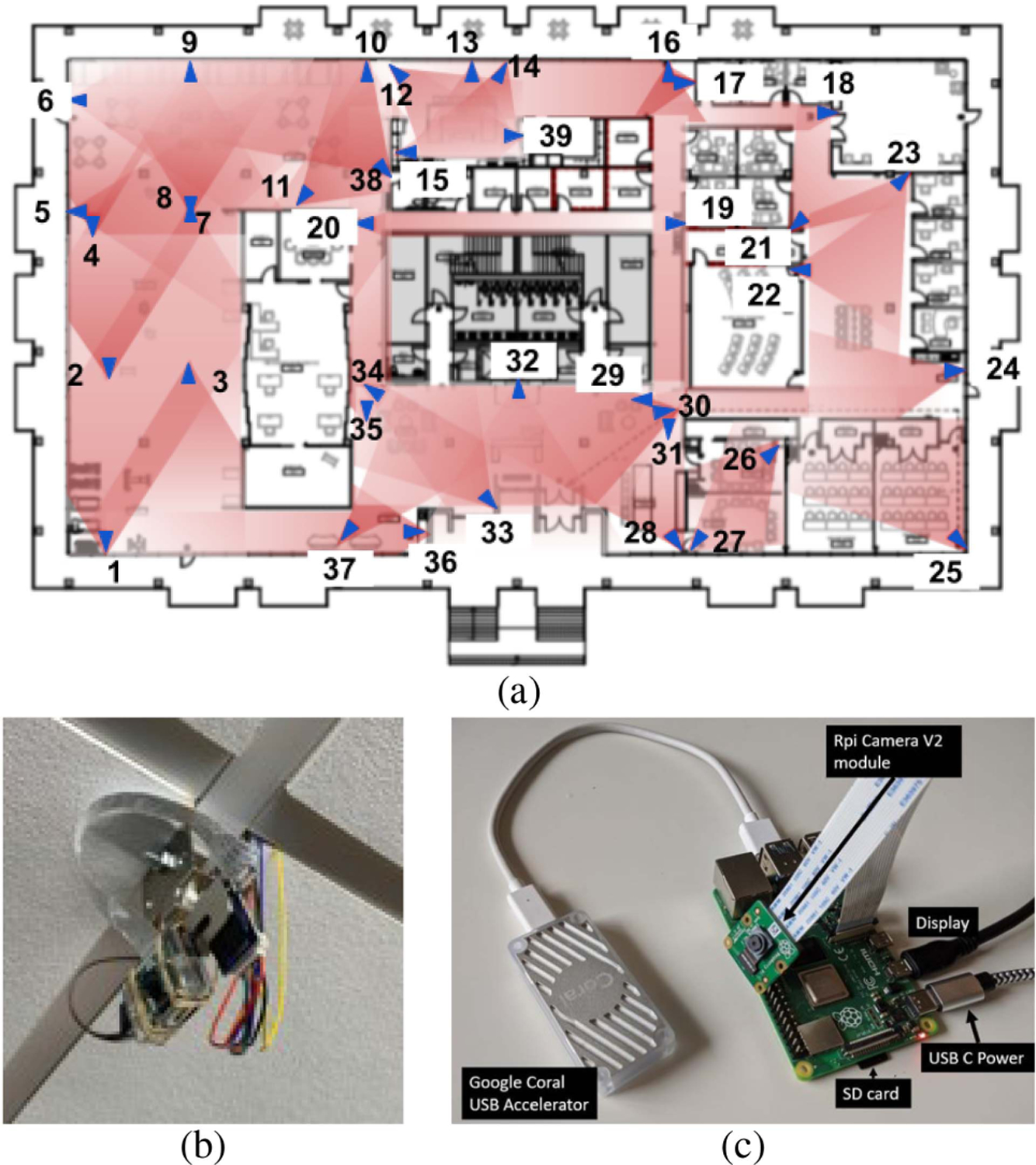
Camera installation in the study site. (a) Field of view (RED) of each camera (Sony IMX219 8-megapixel sensor) is 62.2°, and 39 cameras (BLUE) can cover most regions in our study space. Some regions, such as the kitchen and lounge, are covered by more cameras (five cameras) showing darker red shades. This is due to differences in the number of accessible power and network sources in the ceiling to connect edge computing systems. (b) Camera setup on the ceiling. The edge computing system is concealed in the ceiling and linked to the nearest network and power outlets. (c) Google Coral TPU USB Accelerator enables the edge computing systems to run deep learning models in real time (1 Hz) to detect 2-D poses from videos. The figure is adapted from Kwon et al. [25].

We deployed 39 affordable edge computing camera systems costing less than $200, in our study site as illustrated in Fig. 3(a). These systems consisted of a Raspberry Pi (v4 B), Google Coral USB acceleration device and a Sony IMX219 8-megapixel camera sensor. The field of view [Fig. 3(a), RED] of each camera is 62.2°, and 39 cameras [Fig. 3(b) BLUE] can cover most areas in our study space. Some areas, such as the kitchen and lounge, are covered by more cameras as shown by the darker red shades. The installation of cameras depended on the availability of power and network sources in the ceiling at each location to connect edge computing systems. The cameras were installed on the ceiling panel, and the edge computing devices were concealed in the ceiling panel, as shown in Fig. 3(b).

Multiple cameras were strategically placed in areas such as kitchen and “tech bar” (see Fig. 1) where increased coverage was necessary, while single or dual cameras were sufficient for areas like long corridors. Consequently, this arrangement effectively captured the important spaces within the study site, guaranteeing comprehensive monitoring of subjects’ activities with their informed consent. The camera sensors were connected to an edge computing system that included the Google Coral TPU USB Accelerator, as shown in Fig. 3(c), which enabled running multiperson 2-D pose estimation model [27] in real time (1 Hz) to capture movements while having social activities. The original video frames were discarded, and only the detected poses and bounding box information were kept for further processing to ensure that no personally identifiable information was retained, thereby safeguarding the privacy of individuals.

#### 2) Indoor Localization, Multiperson Tracking, and Body Orientation Estimation:

Kwon et al. [25] processed the detected multiple 2-D poses to obtain multiperson localization, tracking, and body orientation estimation to continuously and passively monitor subjects’ group activities throughout the day. Specifically, the middle point between the left and right feet of 2-D poses from each camera were localized using perspective transformation from 2-D frame space to 2-D floor plane space of the study site. At the same time, the body orientations of the detected individuals were estimated by processing a human bounding box derived from 2-D poses using deep learning models [28]. Duplicated pose detections from overlapping camera viewpoints were integrated using graph analysis. Finally, the multiperson localization across frames was tracked using a Kalman filter-based tracker.

In this work, we aim to determine if the detected locations and orientations of individuals can be successfully used to localize groups across our study space.

### C. Group Identification and Localization

#### 1) Preprocessing Noisy Positions and Orientations:

Lightweight 2-D pose estimation models optimized for resource-constrained edge computing systems produce noisy estimations of both the positions and orientations of individuals. Kwon et al. [25] showed that on average one person in the scene is not detected (false negative) due to occlusions or large distances from the camera. Across frames, at least one false positive (human ghosts) was detected on average due to human-like objects or floor patterns in the study space. To handle false positive detections, we removed all detections where the positions and orientations did not change for a certain amount of time *S* = 10 s, as a person cannot stay absolutely still more than the 10-s period. A person can stay in a certain location for an extended period of time, but orientation is unlikely to stay stationary, which serves as a good indicator of false positive detection of a person. The false negatives (missed detections) will be handled by the following clustering step for group detection.

#### 2) Group Centroid Detection:

From the preprocessed positions and orientations, we used DBSCAN clustering [29] to find clusters of people. DBSCAN is known to be robust to outliers, such as missing samples, and has the ability to detect clusters of arbitrary shapes which is required in this scenario since social groups can have arbitrary formations. Specifically, we use both positions and orientations of the detected individuals with DBSCAN.

We considered that at least two people were needed to form a social group and that people can be as far as 2.1 m to interact. In the theory of proxemics, anthropologist Edward T. Hall studied that most informal business and casual social interactions occurred within a radius of approximately 7 feet (or 2.1 m in SI units) [30]. Beyond this radius, it is more likely that we observe more formal interactions taking place. Since our downstream work focuses on natural informal interactions between individuals, we, therefore, considered social interactions to be taking place when individuals were less than or equal to 2.1 m apart.

**Fig. 4.**
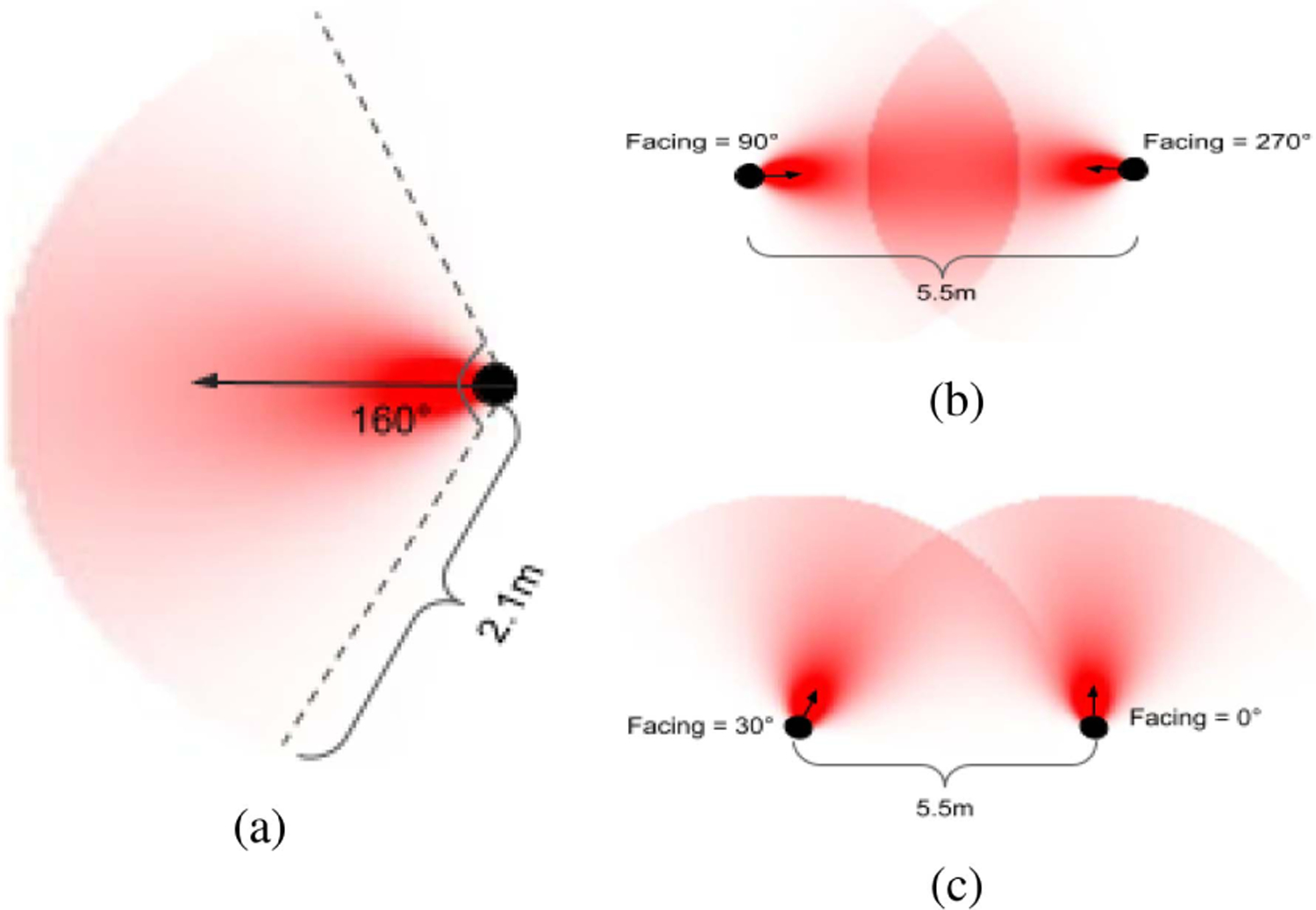
Distance metric calculation using sectors. (a) Sectors obtained by weighting distances up to 2.1 m and angles away from facing angle up to 80° on either side of the individual. These sectors are used to calculate a distance metric between two individuals based on the overlap of sectors of the two individuals. (b) Sectors of two individuals overlapping when they are facing each other at a distance of 5.5 m. (c) Of two individuals overlapping when they are facing almost parallel at a distance of 5.5 m.

With this in mind, we defined the distance metric for DBSCAN as follows. As shown in Fig. 4, we represented the area of social interactions for each individual *i* by a sector of a circle, 𝒜_*i*_, having the radius 2.1 m and angle of 160°. A value of 160° was used as the approximate range of the human eye [31], [32]. We further modeled the intensity of social interaction, *W*, within the sector 𝒜_*i*_ to reciprocally decrease as the distance from the individual increases, and decreases with the distance from the center of facing direction according to Gaussian distribution [32], as

(1) $$W_{d_{i}}=1/L$$

(2) $$W_{\theta_{i}}=N(\theta ,\alpha ,\sigma )$$

(3) $${𝒜}_{i}=W_{d_{i}}+W_{\theta_{i}}$$

where $W_{d_{i}}$ is the weighting based on distance *L* from person *i*’s position. $W_{\theta_{i}}$ is the weighting based on angle away from the facing direction *α* of person *i*, and *σ* is defined as *σ* = 1/3 × 160°/2, following Vascon et al. [32] to ensure 99% of $W_{\theta_{i}}$ in the sector 𝒜_*i*_ remains in the interval [−3*σ*, 3*σ*]. The intensity of interaction between a pair of people, person *j* and person *k*, is defined as the integral of the weights in the overlapping region of their sectors

(4) $$W_{jk}=\int {𝒜}_{j}\cap {𝒜}_{k}$$

Finally, we use the inverse of *W*_*jk*_ as the social interaction distance measured between persons *j* and *k*. This distance is calculated for each pair of people present in the indoor space to form a distance matrix of size *N* × *N*, where *N* is the total number of people detected in the indoor space. DBSCAN uses this distance matrix to cluster people together based on the density of samples (i.e., people). The *ϵ* value for DBSCAN is the maximum distance between two individuals for them to be considered as interacting. To find this, we find the distance metric value as described above for two individuals 2.1 m apart and facing 30° away from each other. Furthermore, in F-formation theory [9], the O-space is in the front of the body at relatively close proximity, where hearing and sight are most effective. We define the center of O-space, or group centroid, as the group location, which is the average position of members in the detected group.

### D. Experiments

#### 1) Benchmark Dataset:

**Fig. 5.**
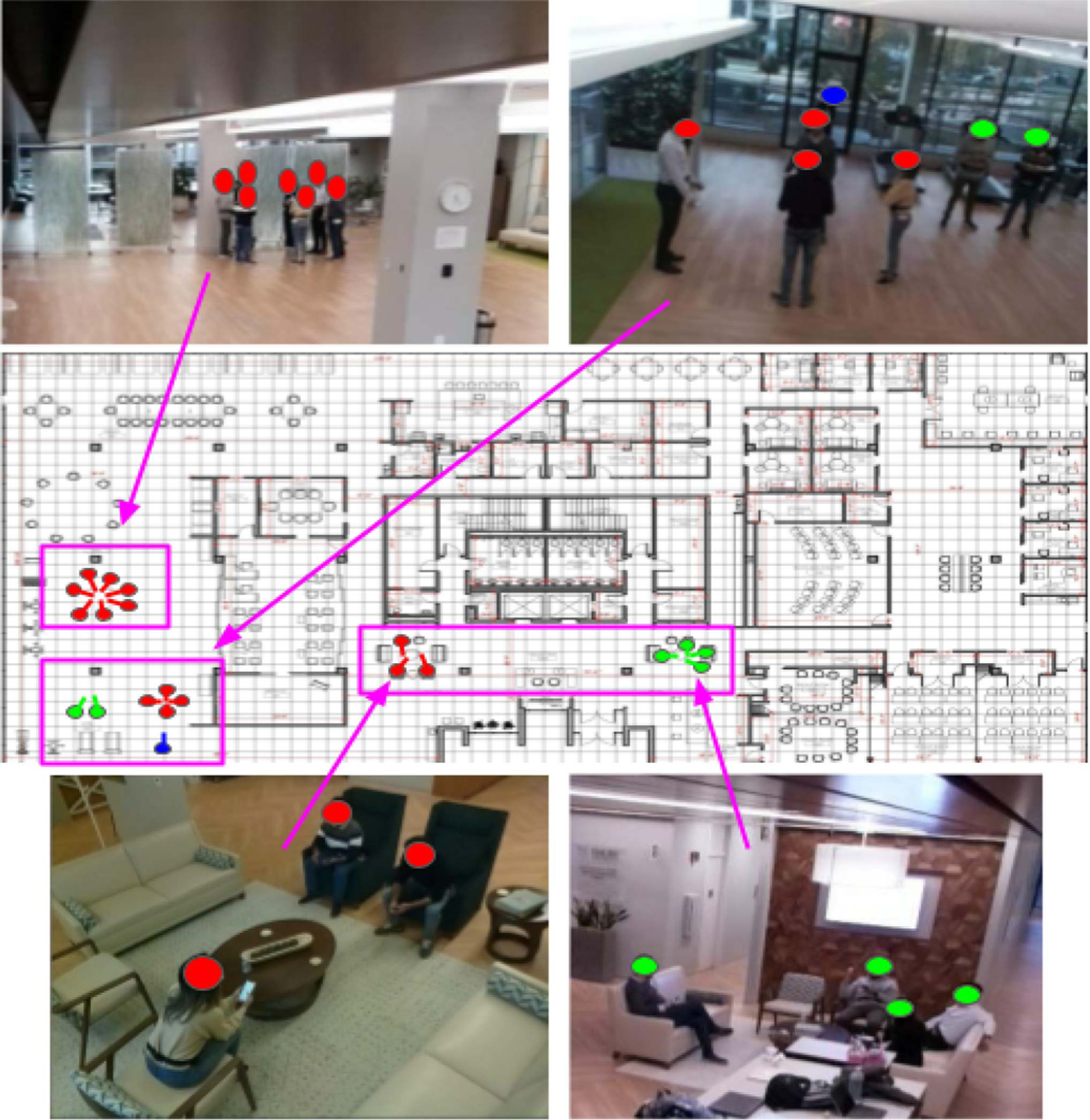
Examples of data collection in the study space with their corresponding ground truth. The images in the top and bottom rows are the raw images captured by the camera. The locations of individuals from camera images are shown on the map in the second row using boxes and arrows. The top left image is taken in the activity area. The top right image is taken in the gym area. The bottom two images are taken in the lounge and tech bar. We simulated realistic group activities often observed from real patients with MCI in our study site. In the map, the circles of the same color depict individuals belonging to the same group. The same colored circles are used in the camera images to show the positions of individuals on the map.

To evaluate our group identification and localization method, we simulated real-world use of the study space and covered instances of group formations of 7 subjects with different sizes while they performed different activities, such as standing, walking, and sitting in our study site of 1700 m^2^. We included group sizes ranging from 2 to 7 people, as well as individuals engaging in activities on their own, in order to assess the capability of our algorithm to detect groups even in the presence of individuals who were not part of those groups. As shown in Fig. 5, the group activities were performed over the entire study space to ensure that different regions with different lighting conditions, camera coverage, and occlusions were considered. The total duration of our data is 35 min, where approximately 5–10 min are spent in each region of the study space. This data collection protocol in each region was performed once and the individuals’ locations and their group formation changed every 30–90 s.

In this work, the social interactions are inferred (i.e., identification and localization of groups) for each second separately. Specifically, following the protocol in Kwon et al. [25], the subjects moved in predetermined paths and group formations for accurate annotation of group activities. The locations and orientations of all the individuals were noted down by an annotator in real time with a margin of error within 1 m, considering different foot sizes and step lengths across individuals and the differences in the actual structure of the study space and the floor plan that was used to develop the localization algorithm. Similar to the previous work focused on dataset collection for indoor localization [33] which relied on regular grids formed on the floor to annotate ground truth locations, the entire study space was taped such that a grid with 1 m × 1 m markings was delineated on the floor. This was used to help with the annotation process to identify the ground truth locations of individuals and group centroids. Consent was procured from subjects to participate in the dataset collection, ensuring informed acknowledgment and agreement.

#### 2) Comparison With Baseline:

We conducted a comparative analysis between the proposed approach and a baseline method that exclusively utilized positional data for DBSCAN clustering, rather than incorporating both positions and orientations. Specifically, we used the Euclidean distance between individuals in the space to detect clusters. Following the proxemics theory [30], we set the parameters of the DBSCAN algorithm as *ϵ* = 2.1 m and *minPts* = 2 (at least two people are needed to form a group). In the subsequent sections, we shall refer to the baseline method, relying solely on positional data as *P* and our proposed method, which incorporates both position and orientation, as *P*+*O*.

#### 3) Evaluation Metric:

In our evaluation, we drew from the insights of proxemics [30] and F-formation theory [9]. We defined the accuracy of group localization based on the proximity of the detected group centroid, which represents the center of the O-space, to the ground truth group centroid. Our benchmark dataset featured multiple groups of varying sizes simultaneously occupying different regions within the study site. To assess the performance of multigroup localization, we initially paired the detected group centroids generated by our method with the closest ground truth group centroids. This matching process was carried out using the Hungarian matching algorithm [34], which is a standard approach in the evaluation of multiobject localization techniques.

For our evaluation criteria, we considered matched centroids with a distance greater than *D* = 3 m as unpaired instances, categorized either as false positives (inaccurate detections) or false negatives (missed detections). This threshold was established based on proxemics theory, which suggests that intimate social interactions occur within a radius of 2.1 m among individuals, with an additional margin of error of 1 m in the ground truth, as explained in Section II-D1. Instances where the matched group locations aligned were deemed true positive detections. This was performed for each frame separately, resulting in a 1-Hz frequency for group identification (since one frame was captured per second).

We report performance scores across various regions within our study site, as illustrated in Fig. 1. This approach allows us to assess our system’s performance in relation to the diverse indoor structures and camera layouts present.

For group identification performance, we compute the average precision, recall, and F1 score using the true positive, false positive, and false negative scores across all samples. The group localization performance is measured by the MAE of the distance between the estimated and ground truth group centroid locations. The overall metrics for the entire study space are obtained by averaging the metrics of each of the regions in the study site.

## III. Results

### A. Group Identification and Localization

Table I shows the precision, recall, F1 score, and MAE for *P* and *P*+*O* for different regions in the study space as well as the average scores across the all study regions. It also shows the approximate space in m^2^ that each region occupies and the number of cameras covering it. *P* showed average precision, recall, F1 score, and MAE of 0.60, 0.57, 0.56, and 1.20 m, respectively, and *P*+*O* showed average precision, recall, F1 score, and MAE of 0.64, 0.55, 0.57, and MAE of 1.25 m for the entire space. Different regions show varying performance for *P* and *P*+*O*. The highest performance is obtained in the lounge area with F1 score of 0.9 and MAE 0.44 for *P*. For *P*+*O*, the highest performance is obtained in the tech bar region with F1 score of 0.89. The precision is generally higher for *P*+*O*. However, the recall is lower compared to *P* which leads to comparable F1 scores. These results show that our method is able to estimate the locations of groups even in the presence of missed and false predictions of positions and orientations for individuals.

### B. Effect of Preprocessing Noisy Positions and Orientations

**Fig. 6.**
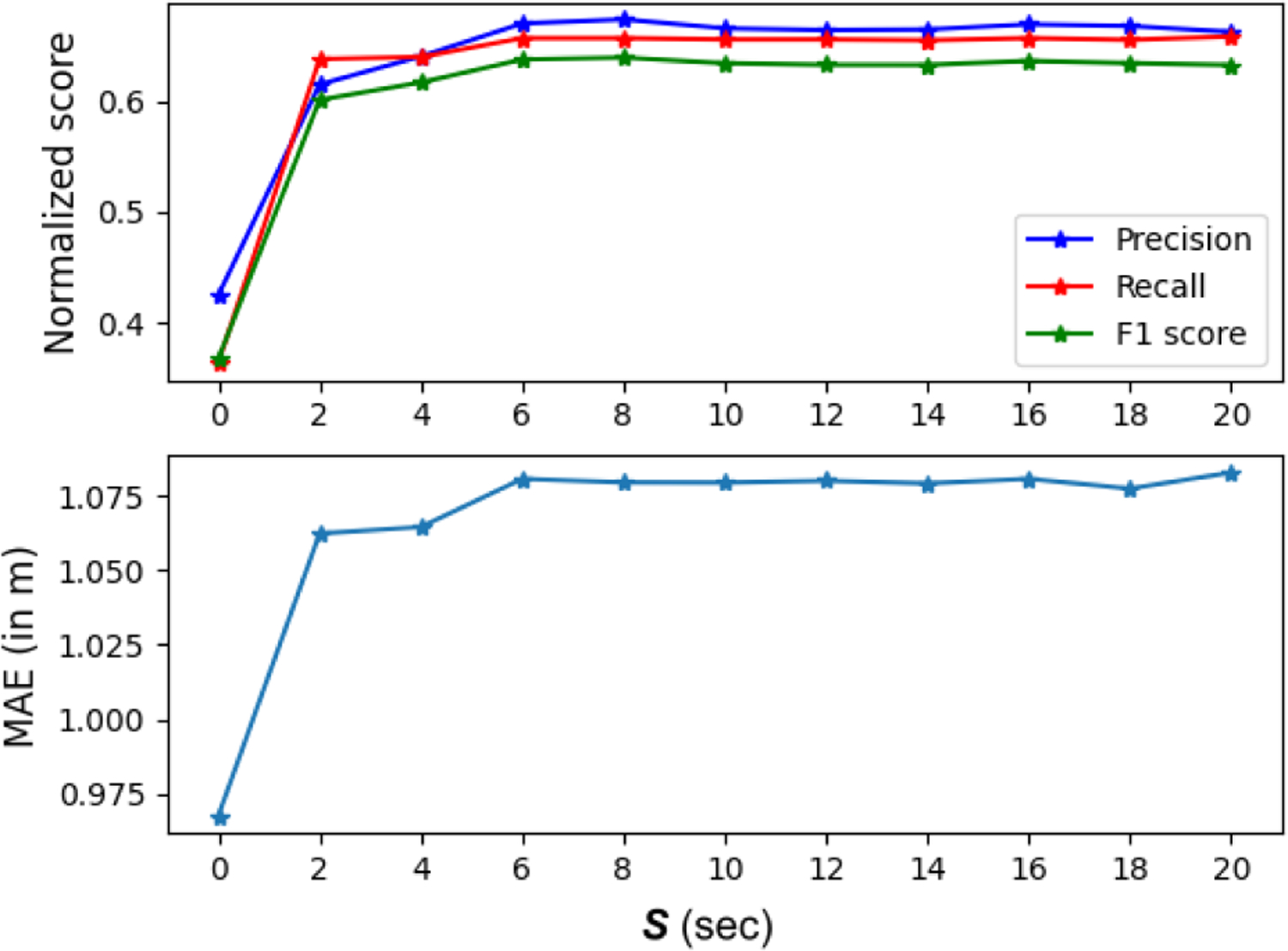
We removed all detections where the positions and orientations did not change for a certain amount of time *S*. This figure shows the precision, recall, F1 score, and MAE as a function of *S*, the preprocessing window, for removing false positive samples. The overall performance increases when *S* increases from 0 to 6 s, and remains relatively flat beyond this point.

Fig. 6 shows how the precision, recall, F1 score, and MAE change for *P*+*O* according to the preprocessing window size for removing false positive samples, 0 *s* ≤ *S* ≤ 20 s as described in Section II-C1. There is a marked improvement in the performance of the algorithm when *S* increases to 6 s.

## IV. Discussion

### A. Group Identification and Localization

Overall, Table I shows comparable performance between *P* and *P*+*O* across varying metrics, but with higher precisions (an absolute increase of 4% on average) for *P*+*O*. The MAE was approximately inversely proportional to F1 score, showing that the location of the identified group is close to the ground truth. We consider this to be due to DBSCAN’s capability to handle outliers, such as false negatives or false positives in clustering samples. This demonstrates that orientation information provides additional information to detect more precise interactions between individuals so that the detected groups are more likely to be true positives than false positives (ghost groups). On the other hand, inaccurately estimated orientation also contributes to result in decreased recall (absolute decrease of 2% on average). We observed that erroneous orientation estimation was more pronounced when individuals were further away from the cameras [25], sometimes detecting people interacting closely as looking away from one another.

**Fig. 7.**
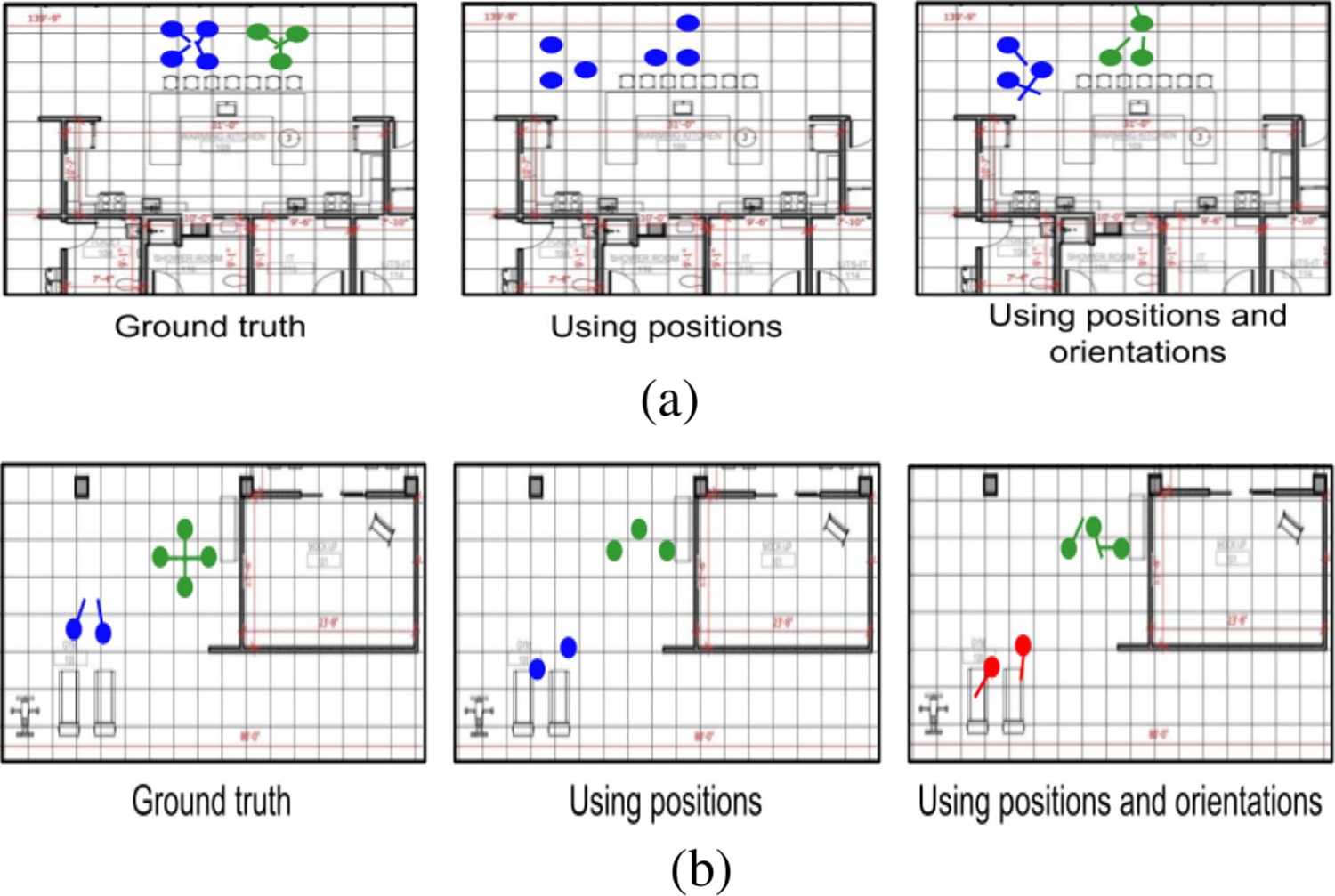
Qualitative analysis comparing ground truth (left) with *P* (middle) and *P*+*O* (right). The dots represent the positions of individuals and the lines represent their orientations. Markers of the same color represent members of the same group. The red markers indicate individuals not part of any group. (a) Example in the kitchen area. *P* (middle), without orientation information, assigns all individuals into one group. *P*+*O* identifies both groups correctly using the orientation information. (b) Example in the gym area. *P* (middle) identifies both groups correctly even though it misses detecting one person in the larger group. *P*+*O* (right) does not identify the group with two people because the estimated orientations of the members of this group are facing away from each other. (a) Kitchen. (b) Gym.

Fig. 7 shows examples of the ground truth groups (left), groups identified using *P* (middle), and *P*+*O* (right) in the (a) kitchen and (b) gym areas. The dots and lines represent the positions and orientations of individuals, respectively. GREEN and BLUE indicate the individuals belonging to same groups and RED indicates individuals not part of any groups. In Fig. 7(a), when only using position information, *P* (middle) identified all individuals as belonging to the same group. But, when orientation information was used in addition to positions, *P*+*O* (right) could correctly identify the two groups.

Fig. 7(b) demonstrates a scenario when inaccurate orientation estimation resulted in wrong group detection. The individuals detected by *P*+*O* (right; REDs) are distant (approximately 8 m) from camera 3 [Fig. 3(a)] and the erroneous orientation estimation prevents *P*+*O* from detecting them as members from the same group. Yet, *P* (middle) could detect those two individuals in the same group (BLUE) based on the individuals positions. This analysis indicates that there is potential for improvement by developing an adaptive approach to integrate position $\left(W_{d_{i}}\right)$ and orientation $\left(W_{\theta_{i}}\right)$ information according to the distance of the detected individuals from cameras in our future work.

Table I also shows the varying performance for *P*+*O* across different regions in our study site. The F1 score is high (0.80 >) for the kitchen, lounge, and tech bar and low (< 0.60) for the gym, activity area, dining area, and staff area. This trend is highly correlated with the density of cameras in these regions. The lounge, with an area of 176 m^2^, is covered by four cameras [cameras 32, 33, 34, and 37 from Fig. 3(a)] with three of them facing toward the sitting area. The tech bar has a smaller area of 66 m^2^ and is covered by two cameras [cameras 28 and 30 from Fig. 3(a)]. The kitchen also has a small area of 70 m^2^ and is covered by five cameras [cameras 10, 13, 14, 15, and 39 from Fig. 3(a)]. Contrarily, the gym, dining, and staff areas cover larger spaces of 168, 147, and 112 m^2^ with only two cameras [cameras 1 and 3 from Fig. 3(a)], three cameras [cameras 6, 8, and 9 from Fig. 3(a)], and three cameras [cameras 21, 22, and 23 from Fig. 3(a)], respectively.

Higher density of the cameras can significantly decrease the distance between individuals and cameras. This results in more accurate position and orientation estimation as well as lower number of false or missed detections of individuals as studied in Kwon et al. [25]. Precise position and orientation estimations lead to more accurate distance calculations between individuals, enhancing the DBSCAN algorithm’s ability to identify clusters(i.e., groups) correctly. Moreover, a reduced occurrence of false or missed detections of individuals leads to lower MAE because there is less significant distortion of estimated group centroids when all group members are successfully detected.

The precision is higher than recall for both *P* and *P*+*O*, suggesting that, on average, there are more false negative than false positive group identifications, i.e., more real groups are missed than phantom (not real) groups are identified. This trend is seen in almost all the regions of the study space except in certain regions with high camera density, such as the kitchen (five cameras covering an area of approximately 70 m^2^). Thus, the performance is greatly affected by missed group identification. This issue can be attributed to the limitations of the 2-D pose estimation algorithm in identifying certain individuals that are occluded or are in the far field. This challenge posed by noisy pose estimations of individuals is further discussed in Section IV-B.

It can be seen that in many regions like the tech bar, kitchen, and lounge, the MAE is greater for *P*+*O* when compared to *P*. This is because the MAE is calculated only for the true positive group identifications, i.e., for matched estimated and ground truth groups locations that are less than 3 m apart. For *P*, there were fewer true positive estimates as compared to *P*+*O*, but these estimates were closer to the ground truth group locations. However, *P*+*O* had a higher number of true positive estimates because it was able to estimate the locations of the groups which *P* missed, within the 3- threshold. This resulted in higher overall MAE. This is also reflected by the higher precision score for *P*+*O*.

The improvements in performance when orientations are considered in addition to positions for group identification and localization are small. In addition, the computational complexity for computing the distance matrix for *P* is *O*(*n*^2^) and for *P*+*O* is *O*(*n*^3^), where *n* is the number of individuals detected in the indoor space. The *O*(*n*^2^) complexity stems from determining distances between every pair of individuals, a shared characteristic for both *P* and *P*+*O*. While calculating the distance between two individuals entails an *O*(1) complexity for *P*, due to Euclidean distance computation, it escalates to *O*(*n*) for *P*+*O* when computing the sector discussed in Section II-C2. However, with improvements in orientation estimation, the performance of *P*+*O* is expected to increase, as suggested by the higher precision rates for *P*+*O*. This improvement can be achieved in several ways, such as increasing camera density, improving the 2-D pose estimation model, or refining the orientation estimation method. Thus, we believe that using orientations for group identification and localization will have increasing benefits. Moreover, it is worth noting that in research scenarios like ours, where real-time group identification and localization are not the focus, optimizing computational complexity becomes less pressing. In such contexts, the advantages of incorporating orientations outweigh the limitations posed by computational complexities.

### B. Challenges From Noisy Detections of Individuals

**Fig. 8.**
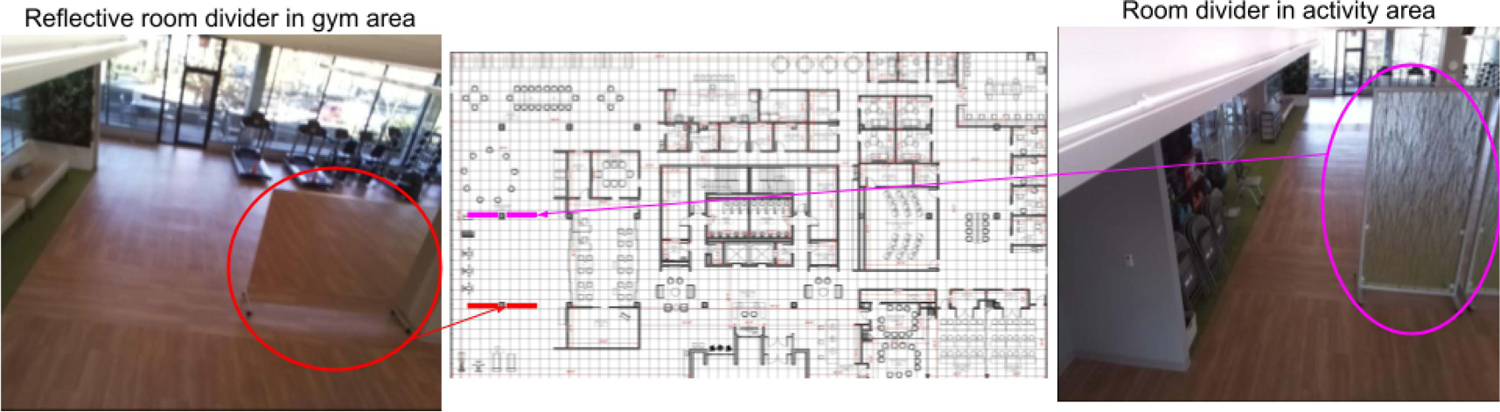
Examples of room dividers placed in the built environment. The image on the left shows a reflective room divider placed in the gym area and the image on the right shows a nonreflective room divider placed in the activity area. The positions of these room dividers are shown on the map in the middle image. The presence of the room dividers cause occlusions, thus, resulting in some people not being picked up by the cameras, leading to false negative detections of individuals which affects group localization.

We encountered distinct challenges in our group localization efforts, primarily stemming from occlusions present in complex spaces. Specifically, the gym and activity areas, which collectively occupy 312 m^2^, are surveilled by seven cameras [cameras 1, 2, 3, 4, 5, 7, and 8 as depicted in Fig. 3(a)]. Nevertheless, these spaces are equipped with room dividers, both reflective and nonreflective, that obstruct visibility, as illustrated in Fig. 8. In addition, the dining and staff areas are densely furnished with tables and chairs, some of which obscure individuals’ lower bodies or even their entire figures, contingent on the camera’s perspective. These occlusions present formidable challenges, resulting in instances of missed detections of individuals or the erroneous detection of human-like objects in the environment, akin to “human ghosts.” Consequently, these issues diminish the accuracy of group identifications.

These structural disparities among the various regions also manifest in distinct characteristics in the estimated positions and orientations, as detailed in Kwon et al. [25]. These variations can significantly influence the performance of the DBSCAN clustering. The performance of DBSCAN is sensitive to the *ϵ* parameter. Therefore, an enticing avenue for future research lies in establishing adaptive hyperparameters for DBSCAN tailored to each specific region within the study space.

While we effectively managed false positives during the preprocessing stage, as evidenced in Fig. 6, false negatives remain a formidable challenge. DBSCAN clustering is capable of identifying groups with missing members. However, if a substantial portion of group members goes undetected, it is highly likely that the group will not be detected. Moreover, when multiple members within a large group remain undetected, the calculated centroid of the group may skew toward the region where more members were detected, resulting in an increased MAE. These errors are exacerbated by a lower number of cameras in certain regions and the increased distance of individuals from the cameras. This results in missed detections of some individuals. Furthermore, this leads to relatively more detections occurring at the edge of the field of view of the cameras, resulting in distorted position and orientation estimations of individuals which affect the group localization process. The effects of lower camera density on the position and orientation estimation of individuals are discussed in detail by Kwon et al. [25].

In our forthcoming work, we intend to explore diverse multiperson pose estimation models [35] and model compression techniques [36] suitable for resource-constrained computing systems like ours. We will also explore increasing the camera densities based on findings from both [25] and this work. This endeavor aims to address the aforementioned challenges and further enhance our system’s performance. We also aim to explore the effects of using different values for the maximum distance between two people to be considered an interaction, which is currently 2.1 m, on the performance of the proposed algorithm and the kinds of social interactions observed.

### C. Potential Applications for Health Analysis

The proposed group identification and localization system designed for built environments represent a crucial initial stride toward unraveling the intricacies of indoor space utilization and the social interaction patterns of individuals dealing with cognitive impairment. This exploration encompasses the following fundamental inquiries.

1. Frequency of social interaction: Which indoor regions witness more frequent social interactions?
2. Spatial arrangement impact: How do alterations in the spatial layout influence social interactions?
3. Temporal patterns: Are there specific times of the day when social interactions are more prevalent?

We plan to study this series of questions focusing initially on regions where the group localization system has demonstrated high accuracy. Notable among these are the kitchen, lounge, and tech bar zones. For other regions, our objective is to extend camera coverage to enhance group localization performance, contingent on the availability of power and network resources in the ceiling. Moreover, owing to the cost-effectiveness and scalability of our proposed system, we envision its deployment in numerous therapeutic units catering to diverse populations coping with mental and cognitive challenges, including conditions like autism and bipolar disorder [37], [38], which are known to significantly impact social behaviors of individuals.

## V. Conclusion

Designing a smart therapeutic space that can passively and continuously monitor the movements of individuals in a privacy-preserving manner provides a new opportunity to assess patients’ mental and cognitive health. To this end, we propose a group localization technique that is applicable to distributed cameras installed with edge computing platforms. Our study space poses significant challenges for identifying individuals and groups due to its wide space spanning 1700 m^2^ and complex structures having room dividers, mirrors, or furniture. Nonetheless, the proposed system could localize multiple groups with F1 score of up to 90% and MAE as low as 0.44 m depending on the camera coverages and structure of the regions, even with the presence of noisy position and orientation estimations of individuals from lightweighted multiperson pose estimation models.

In our forthcoming research endeavors, we intend to explore an adaptive group localization approach that factors in camera distances from individuals and region-specific clustering models. This tailored approach takes into account the distinct complexities inherent in each region within our study space. To facilitate these endeavors, we have made the source code [39] of the proposed system available to encourage practitioners to transform any ordinary therapeutic space into the smart environments to monitor behaviors related to mental and brain health.

## Figures and Tables

**TABLE I:** Precision, Recall, F1 Score, and MAE for Localizing Groups in Different Regions in the Built Environment

			Position-based group localization *(P)*				Position + orientation-based group localization (*P+O*)			
Region	# 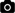	Area (*m*^2^)	Precision	Recall	F1 score	MAE (*m*)	Precision	Recall	F1 score	MAE (*m*)

Gym	2	168	0.59	0.49	0.52	1.26	0.65	0.48	0.53	0.98
Activity area	6	144	0.46	0.46	0.46	1.35	0.46	0.46	0.46	0.87
Dining area	3	147	0.59	0.40	0.45	1.36	0.62	0.34	0.42	1.22
Kitchen	5	70	0.71	0.84	0.73	0.90	0.81	0.83	0.81	1.85
Lounge	4	176	0.91	0.92	0.90	0.44	0.91	0.86	0.85	0.50
Tech bar	2	66	0.94	0.89	0.89	1.08	0.99	0.84	0.89	1.16
Staff area	3	112	0.03	0.03	0.03	2.05	0.05	0.05	0.05	2.19

Overall	–	–	0.60	0.57	0.56	1.20	0.64	0.55	0.57	1.25

Number of cameras in each region (#) and area covered by each region (Area) are also shown. Different regions (per Fig. 1) have different performances which are due to various factors like occlusion, distance from camera, number of cameras and relative camera coverage in the region.
